# Flow Pressure Characteristics of the Ahmed Glaucoma Valve and Possible Effect of Entrapped Air in the Tube

**DOI:** 10.1167/tvst.12.4.16

**Published:** 2023-04-14

**Authors:** Andi Masdipa, Sachiko Kaidzu, Masaki Tanito

**Affiliations:** 1 Department of Ophthalmology, Shimane University Faculty of Medicine, Izumo, Japan

**Keywords:** priming pressure, flow rate, water hammer, newton's second law, Bernoulli's principle

## Abstract

**Purpose:**

The purpose of this study was to assess the pressure characteristics of the Ahmed Glaucoma Valve (AGV) and possible effects of air trapped in the tube.

**Method:**

Physiologic saline was pumped through 17 AGVs using a syringe infusion pump, and the flow pressure was measured by a set of pressure transducers. During the infusion at a rate of 2 µL/minute, the pressure measurement was repeated twice in each AGV to determine the repriming pressures with/without air (1 µL) in the tube.

**Results:**

After a pressure surge occurred during the initial priming, the pressure decreased suddenly and then became constant. The repriming pressure, determined as the peak pressure before valve opening, was significantly (*P* < 0.0001, paired *t*-test) higher with air (26.5 ± 6.8 mm Hg) than without air (12.1 ± 3.8 mm Hg), whereas the constant pressures after repriming was equivalent between with (10.6 ± 3.7 mm Hg) and without (10.4 ± 2.9 mm Hg) air conditions (*P* = 0.68).

**Conclusions:**

Air in the AGV tube causes increased repriming pressure of about two-fold compared to repriming without air. This pressure increment caused by air in the capillary-sized tube might occur because of the effects of viscosity pressure and capillary pressure.

**Translational Relevance:**

To ensure stable surgical results**,** surgeons are advised to not allow air to remain in the tube. Pars plana tube insertion of the AGV combined with gas tamponade surgery may result in higher-than-expected intraocular pressure. Conversely, injection of air/gas can avoid postoperative hypotony when the AGV is implanted in eyes with a high risk of hypotony.

## Introduction

Glaucoma is the second leading cause of blindness worldwide after cataracts.^1^ In 2040, an estimated 111.8 million people will have glaucoma.^2^ The main goal of treating glaucoma is to reduce intraocular pressure (IOP) to a level that slows disease progression. Surgery is an appropriate therapeutic option to treat glaucoma in cases refractory to pharmacologic treatment. Tube shunt surgery using the Ahmed Glaucoma Valve (AGV) is an option that surgeons frequently choose.^3^^–^^5^ AGV-related complications, such as hypotony and hypertension, have been widely reported, although the AGV is associated with a lower chance of hypotony than hypertension because of its valve mechanism.^6^^,^^7^ Several in vitro studies have determined the pressure required to open the valve of the device (referred to as priming) when it was first used.^8^^,^^9^ However, the flow characteristics after the initial priming have not been fully tested. Initial priming is done intra-operatively by injecting about 1 cc of ophthalmic irrigation fluid (e.g. balanced salt solution) using a 26-gauge (G) or 27-G needle.^1^ When the needle is removed from the AGV tube, the part that previously contained the needle no longer contains fluid; thus, a small amount of air is frequently trapped in the tube after the priming. This study assessed the pressure characteristics of the AGV after initial priming and the possible effects of trapped air through an experimental infusion system.

## Materials and Methods

**Figure 1.**
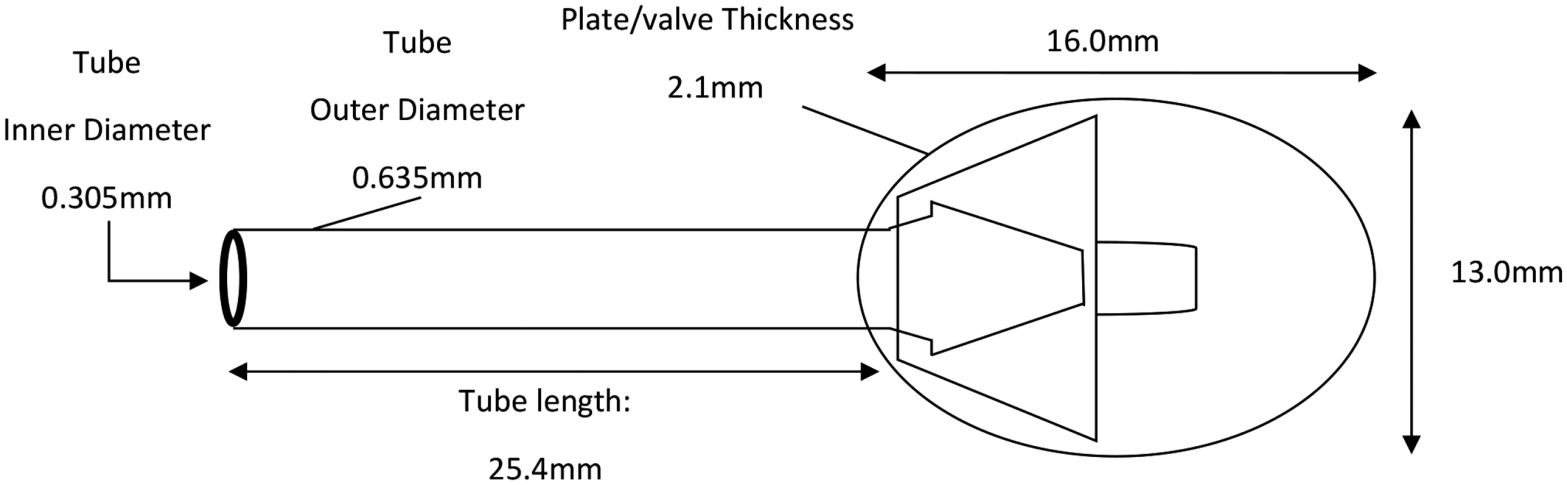
Specifications of the AGV FP7.

Seventeen AGV devices (model FP7; New World Medical, Rancho Cucamonga, CA) were tested in this study. The AGV specifications are shown in Figure 1. Physiologic saline (0.7% sodium chloride), disposable 27-G needles (NN-2719S), and 5-mL syringes (SS-05Sz) were purchased from Terumo Corporation (Tokyo, Japan). Infusion tubes (JV-NDH1050FL and JV-ND1010PC) were purchased from JMS Co., Ltd. (Tokyo, Japan). An infusion syringe pump (SP101i) was purchased from Kd Scientific (Holliston, MA). A pressure transducer (BLPR2), 4-channel transducer amplifier (SYS-TBM4M), analog-to-digital converter (LAB-TRAX-4/16), and pressure curve analysis software LabScribe2 (LAB-TRAX-4) were purchased from World Precision Instrument (Sarasota, FL).

### Experimental Settings

**Figure 2.**
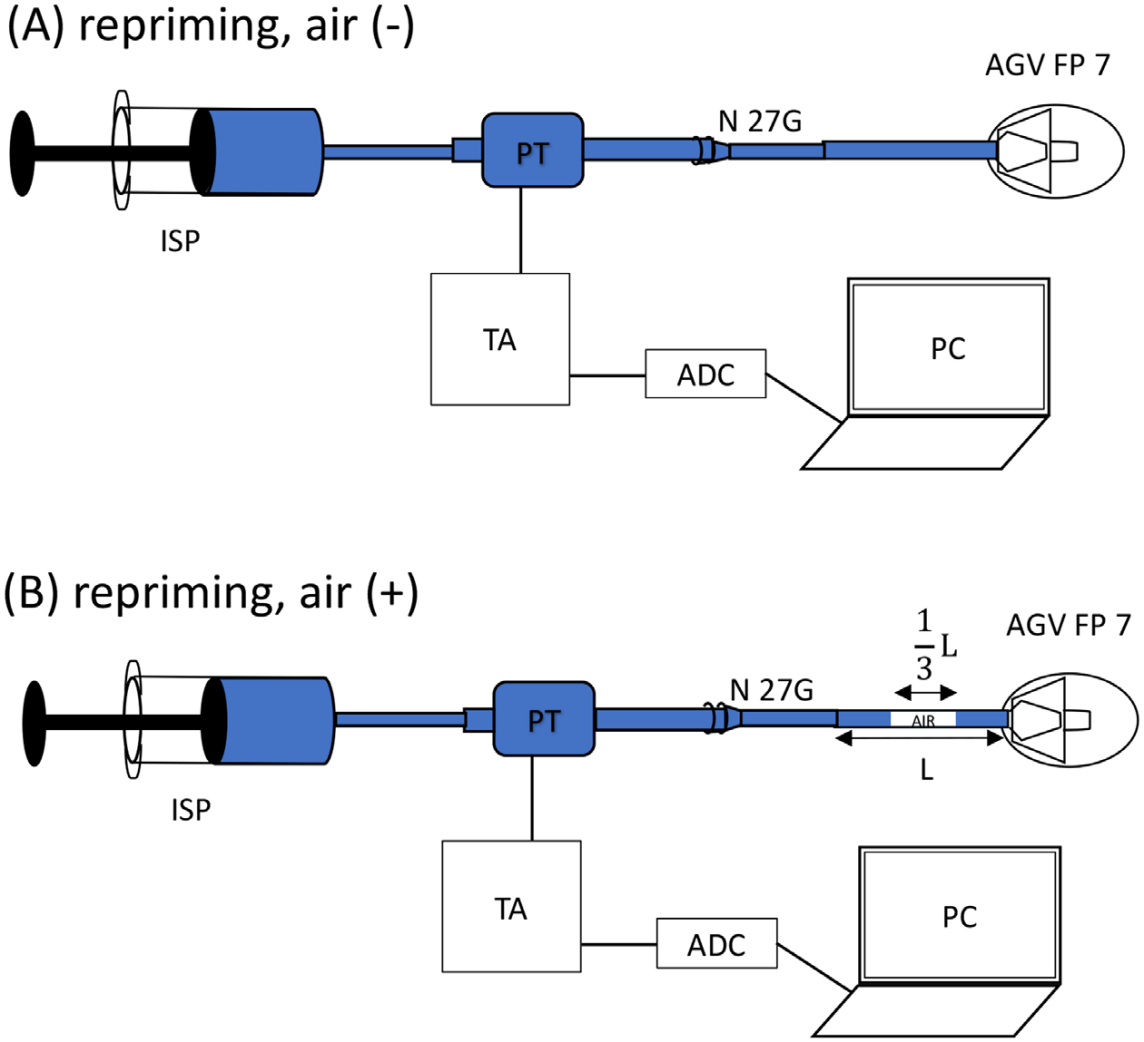
Configuration of the pressure measurements without (**A**) and with (**B**) air in the tube. ISP, infusion syringe pump; PT, pressure transducer; TA, transducer amplifier; ADC, analog-to-digital converter; PC, personal computer; L, tube length; AGV, Ahmed Glaucoma Valve.

Figure 2 shows the schematic setting of the experiment. A 5-mL syringe attached to the infusion syringe pump facilitated flow of the physiologic saline to the pressure transducer with an infusion tube, and then from the pressure transducer, the physiologic saline flowed into the AGV through a 27-G needle that was connected to the AGV tube (see Fig. 2A). The pressure transducer was connected to a personal computer through the transducer amplifier and an analog-to-digital converter. To assess the roles of air trapped in the tube, about 1 µL of air was injected into the middle of the AGV tube at about one-third of the length of the tube (see Fig. 2B). Before connecting the AGV to the 27-G needle, the transducer amplifier was calibrated so that the pressure value in the LAB-TRAX4 software showed that the pressure value was zero.

After the AGV tube was connected to the 27-G needle, the injection of the physiologic saline began at a flow speed of 100 µL/minute to fill the previously unprimed AGV tube. The pressure increased to the peak pressure, referred to as the initial priming pressure, and then dropped (Fig. 3①). The flow was stopped after the initial priming occurred, which was marked by the release of physiologic saline flowing through the AGV valve. Air (1 µL) was injected into the AGV tube, and the measurement was continued at a flow rate of 2 µL/minute. The pressure continued to increase until it reached the peak, which was recorded as the pressure of repriming air (+) (see Fig. 3②). The pressure decreased and became constant; this was recorded as the constant pressure after repriming air (+) (see Fig. 3③). After the flow was stopped for about 15 minutes, the flow was restarted at 2 µL/minute. The pressure continued to increase to the peak pressure, which was recorded as the pressure of repriming air (−) (see Fig. 3④). The pressure then decreased and became constant, and this was recorded as the constant pressure after repriming air (−) (see Fig. 3⑤).

**Figure 3.**
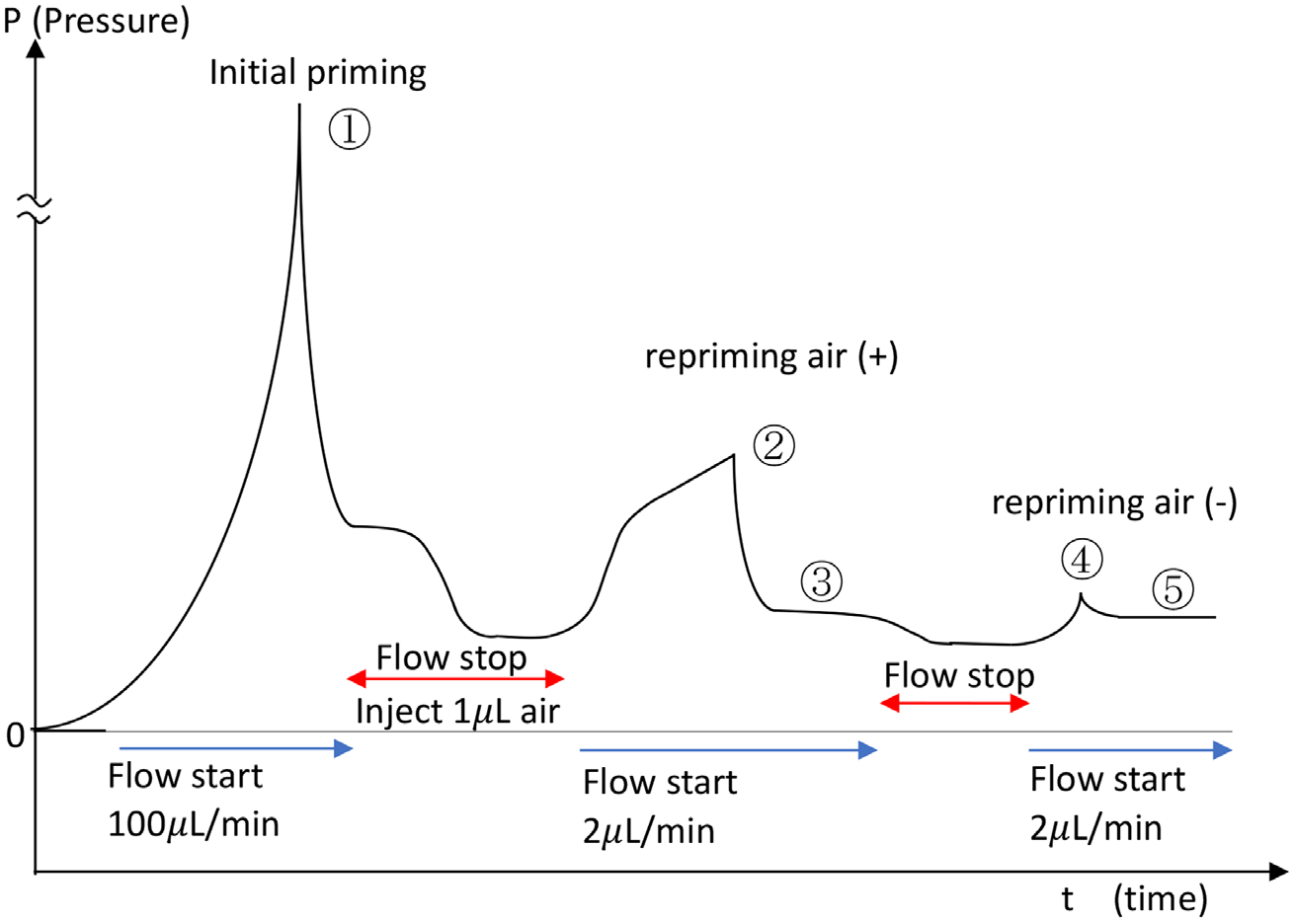
Graphic illustration of the pressure characteristics of AGV during the pressure measurements. ① Initial priming pressure; ② repriming pressure, air (+); ③ constant pressure after repriming, air (+); ④ repriming pressure, air (−); and ⑤ constant pressure after repriming, air (−).

### Data Analysis

Based on the information provided by the manufacturer, the inspection standards for AGV have changed since June 2020. At that time, the inspection standard for the valve closing pressure of 5.1 to 9.3 mm Hg previously was changed to 6.00 to 13.00 mm Hg. Therefore, we also compared the pressure characteristics between old lots (7 old types) and new lots (10 new types). The data were analyzed using JMP Pro 16 statistical software (JMP Statistical Discovery, Cary, NC). All data are expressed as the mean value ± standard deviation. Comparisons between air (+) and (−) conditions were tested using the paired *t*-test and between new and old lots using the unpaired *t*-test.

## Results

Five pressure characteristics (see Fig. 3) were measured in each AGV. In several devices, the initial priming pressure (corresponding to Fig. 3①) values exceeded the measurement scale; the other 4 pressure characteristics were obtained successfully in all 17 devices.

**Figure 4.**
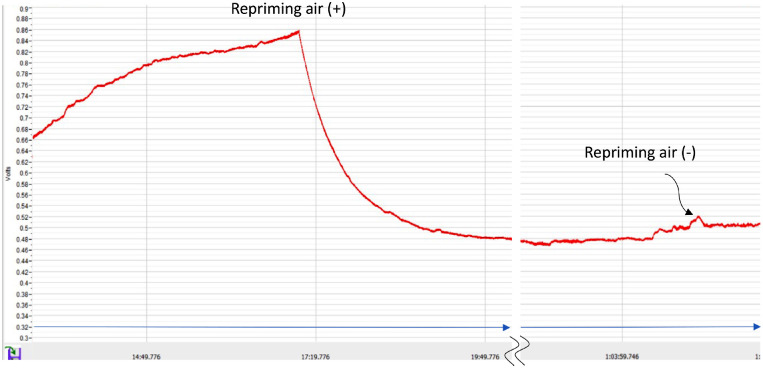
Representative recordings of the flow pressures in the air (+) and (−) repriming conditions.

Representative recordings of the flow pressures in the air (+) and (−) repriming conditions are shown in Figure 4. Unexpectedly, the pressure peak in the air (+) condition was markedly higher than in the air (−) condition (see Fig. 4). The measured repriming pressure and constant pressure after repriming with/without air are summarized in Table 1. The mean value of the repriming pressures in the air (+) condition (26.5 mm Hg; see Fig. 3②) was significantly (*P* < 0.0001) higher than that in the air (−) condition (12.1 mm Hg; see Fig. 3④). In both conditions, the constant pressure after repriming was lower than the repriming pressure (*P* < 0.0001 for both conditions). In contrast to the repriming pressures, the constant pressures after repriming with air (10.6 mm Hg; see Fig. 3③) and without air (10.4 mm Hg; see Fig. 3⑤) were equivalent (*P* = 0.68).

**Table 1.** Comparisons in Measured Pressures Between Air (+) and (−) Conditions (*N* = 17)

Pressure (mm Hg)	Air (+)	Air (−)	*P* Value, Air (+) vs. (−)
Repriming	26.5 ± 6.8	12.1 ± 3.8	<0.0001
Constant after repriming	10.6 ± 3.7	10.4 ± 2.9	0.68
*P* value, repriming versus constant after repriming	0.009	<0.0001	

*P* values were obtained using the paired *t*-test.

Table 2 summarizes the measurement values stratified by new and old AGV lots. The repriming pressure with air was higher in old lots (30.5 mm Hg) than new lots (23.8 mm Hg), a difference that reached borderline significance (*P* = 0.04), whereas there was no significant difference in other comparisons.

**Table 2.** Comparisons in Measured Pressures Between AGVs From New and Old Lots

Pressure (mm Hg)	New (*n* = 10)	Old (*n* = 7)	*P* Value, New Versus Old
Repriming			
Air (+)	23.8 ± 1.9	30.5 ± 2.3	0.04
Air (−)	12.3 ± 3.6	11.9 ± 4.2	0.82
Constant after repriming			
Air (+)	9.8 ± 2.7	11.7 ± 4.8	0.33
Air (−)	10.3 ± 2.9	10.6 ± 3.2	0.86

*P* values were obtained using the unpaired *t*-test.

## Discussion

In this study, measurements of the pressure characteristics of the AGVs were performed in vitro, and five flow pressure characteristics of the AGV were assessed. The results indicated that the pressure of the repriming air (+) was twice that of the pressure of the repriming air (−), but constant pressures were equivalent between both conditions.

The initial priming pressure was the amount of pressure required to open the AGV valve when it was first used. According to the manufacturer's instructions, the initial priming should be performed by injecting 1 mL of physiologic saline.^1^^,^^6^^,^^9^ In addition to opening and reducing the valve resistance in the AGV before its implantation, another purpose is to remove air in the device and ensure that there are no manufacturing defects.^8^ Cheng et al.^9^ measured this priming pressure and reported an average of 3000 mm Hg; even with priming 3 times, the pressure greater than priming did not damage the function of the silicone-plate valve of the AGV.^9^ In our experiments, the initial priming pressure exceeded the measurement scale in some devices; thus, the observation reproduced the previous report that showed an extremely high initial priming pressure.

The function of the AGV valve is to maintain the pressure to prevent postoperative hypotony.^4^ When the flow of physiologic saline was stopped or decreased drastically, the valve closes to prevent the liquid from continuing to flow out, so that the pressure at the inlet tube is maintained at a certain pressure. Based on previous research, the closing pressure on the AGV was around 10 mm Hg.^4^ In the current study, when physiologic saline was reflowed at the same volume rate, the pressure transducer showed that the pressure continued to increase to the peak to reopen the valve that was previously closed. We referred to this peak pressure as the pressure of repriming air (−) (see Fig. 3④). The increased pressure in this state was caused by the closing of the valve and the increased volume of fluid as the rate of physiologic saline continuing to flow until the valve reopened. This pressure increase can be explained by the water hammer equation approach on the pipeline. The following is a description of Equation 1^10^:

(1) $$\begin{array}{c} c=\frac{1425}{\sqrt{1+\frac{2,1\;\times 10^{4}d}{E\partial }}} \end{array}$$

where с is the pressure wave propagation (increased and decreased pressure) in a pipeline (m/s); where ∂ (pipeline wall thickness) = 1.65  × 10^−4^ *m*, *d* (inner diameter) = 3.05  × 10^−4^ *m*, *E* (module of elasticity) = 1  × 10^6^ *N*/*cm*^2^, Δ*v* (velocity diffrence) = 4.56  × 10^−4^ *m*/*s*, and ρ (density of water) = 1000 *Kg*/*m*^3^.

(2) $$\begin{array}{c} \Delta P=\rho \left(\frac{c}{g}\right)\Delta v \end{array}$$

where Δ*P* is the pressure difference between *P*_1_ and *P*_2_ (*P*_1_ − *P*_2_)

(3) $$\begin{array}{c} P_{1}-P_{2}=\frac{1425\;\rho \Delta v}{g\sqrt{1+\frac{2.1\;\times 10^{4}d}{E\partial }}\;} \end{array}$$

(4) $$\begin{array}{c} P_{1}-P_{2}=64.98\;Pa=0.487\;mm\;Hg \end{array}$$

Equations 2 and 3 served as approximations to explain the increasing pressure on repriming. By entering the approximate value of each variable, the value of the pressure difference was calculated to be 0.487 mm Hg, as in (Equation 4). This value is even smaller than the measured pressure difference between the repriming pressure and the constant pressure after repriming in the air (−) condition (i.e. 12.1–10.4 =1.7 mm Hg), and may indicate the presence of other factors that affect the magnitude of the increase in the repriming pressure (e.g. resistance derived from valve stiffness).

**Figure 5.**
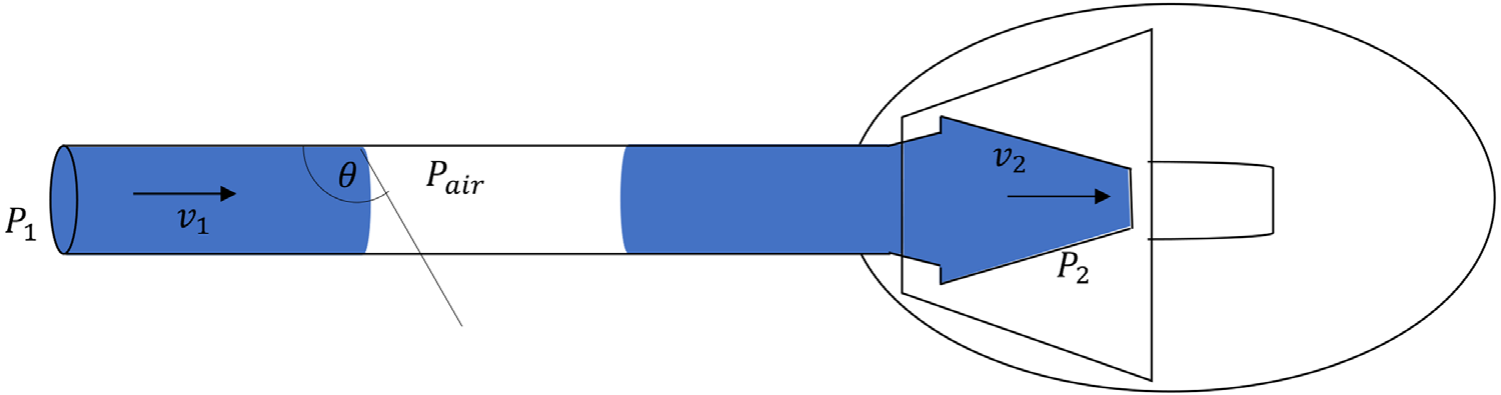
The flow dynamics of the AGV in the air (+) condition. *P*_1_, the pressure measured at the pressure transducer; *P_air_*, air pressure; *P*_2_, pressure at the valve; *v*_1_, fluid velocity at the tube input; *v*_2_, fluid velocity at the valve; and θ, contact angle.

The above equations describe the air (−) condition, and the measurement results indicated that the pressure of the repriming air (+) condition was about two times higher than that of the repriming air (−) condition; thus, the trapped air in the tube became an additional factor that increased the valve resistance. The pressure of the repriming air (+) was measured as in Figure 5, in which the measured pressure was *P*_1_. The increase in pressure *P*_1_ can be explained by complex fluid dynamics because the role of the air in the tube was affected by various factors, including the tube diameter and material. Given that the inner tube diameter was in micrometers, the flow was associated with the capillary phenomenon and surface tension. This capillarity phenomenon also was affected by the cohesion force (attraction between similar particles) and the adhesion force (attraction between different types of particles). In addition, AGV FP7 is made of medical grade silicone, which is water-repellent.^11^^,^^12^ This water-repellent characteristic made the cohesion force exceed the adhesion force.^12^^,^^13^ That scenario makes the surface of the liquid a convex meniscus so that the fluid in the tube forms a larger angle contact (θ > 90°).^13^ Equation 5 for capillary pressure and its relationship to the surface tension in the pipe tube is as follows^14^^–^^16^:

(5) $$\begin{array}{c} P_{capillary}=\frac{2\gamma \cos\theta }{r} \end{array}$$

In addition to capillary pressure, fluid viscosity also affects high pressures, with the drag force of fluid viscosity as follows in (Equation 6)^14^^,^^15^:

(6) $$\begin{array}{c} F_{v}=8\pi l\eta v \end{array}$$

where

(7) $$\begin{array}{c} P_{v}=\frac{F_{v}}{A}=\frac{8\pi l\eta v}{\pi r^{2}} \end{array}$$

and

(8) $$\begin{array}{c} P_{v}=\frac{8\eta lv}{r^{2}} \end{array}$$

Pressure Equation 7 was obtained by dividing the drag force Equation 6 into the cross-sectional area (A = π*r*^2^). Equation 8 then was a simplification of Equation 7, namely, the pressure equation by drag force. Based on Newton's second law, the equation of the motion of the meniscus liquid is as follows:

(9) $$\begin{array}{c} \left(P_{1}-P_{2}-P_{air}\right)\pi r^{2}=\;8\;\pi l\eta v-2\;\gamma \pi r\cos\theta \end{array}$$

(10) $$\begin{array}{c} P_{1}-P_{2}=\;P_{air}+\frac{8\eta lv}{r^{2}}-\frac{2\gamma \cos\theta }{r} \end{array}$$

In Equation 9, after dividing both sides by the cross-sectional area of the tube (π*r*^2^), the pressure difference (*P*_1_ − *P*_2_) is expressed as in Equation 10. *P*_1_ is the pressure measured at the pressure transducer, *P_air_*, air pressure; η, fluid viscosity; γ, surface tension; *v*, fluid velocity; *l*, tube length; *r*, inner radius of the tube; and θ, contact angle. In Equation 10, the value of *P*_1_ was determined by the air pressure plus the viscosity pressure and minus the capillary pressure. Because of the characteristics of the tube material (θ > 90°), the cos θ is negative. Mathematically, capillary pressure should reduce the value of *P*_1_, but the minus sign (−) on the capillary pressure when multiplied by the minus (−) value of cos θ produces a positive value, which will make the value of *P*_1_ increase. Accordingly, capillary pressure and drag force should explain in part the effect of the trapped air on the increase in the repriming pressure rise.

**Figure 6.**
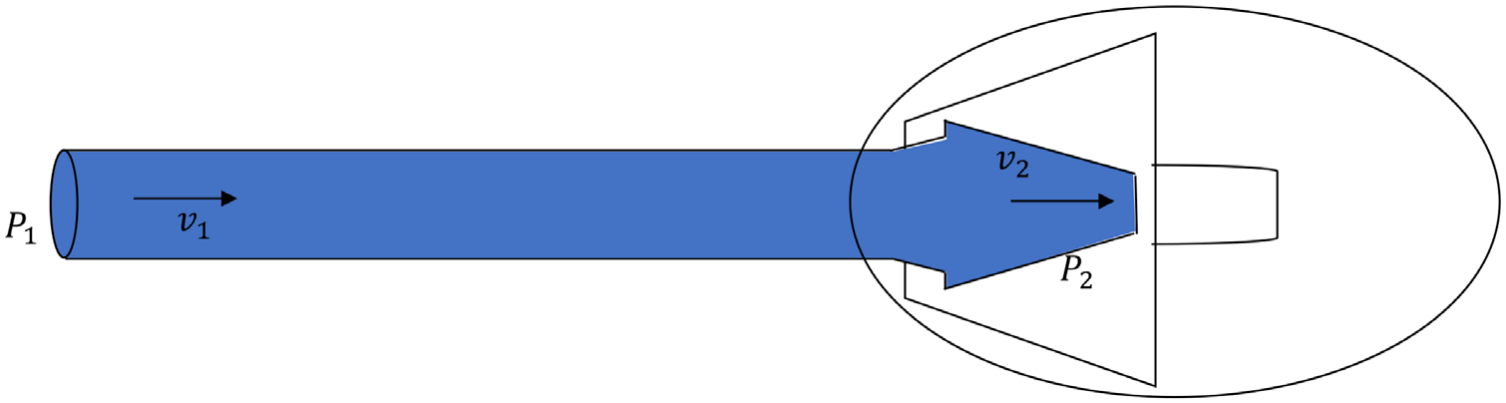
The flow dynamics of the AGV in the air (−) condition. *P*_1_, the pressure measured at the pressure transducer; *P*_2_, pressure at the valve; *v*_1_, fluid velocity at the tube input; and *v*_2_, fluid velocity at the valve.

After priming, the AGV works as a drain where the fluid will flow through the AGV tube and then flow out through the valve. In this condition (Fig. 6), the AGV works as a venturi with Bernoulli's principle as in the following Equation 11^1^:

(11) $$\begin{array}{c} P_{1}+\frac{1}{2}\rho v_{1}^{2}+\rho gh_{1}=P_{2}+\frac{1}{2}\rho v_{2}^{2}+\rho gh_{2} \end{array}$$

(12) $$\begin{array}{c} P_{1}=P_{2}+\frac{1}{2}\rho \left(v_{2}^{2}-v_{1}^{2}\right)+\rho g\left(\Delta h\right) \end{array}$$

Equation 12 was obtained after simplifying Equation 11, the heights of *h*_1_ and *h*_2_ were considered equal in our setting, so that Δ*h* = *h*_2_ − *h*_1_ = 0 and then obtained Equation 13:

(13) $$\begin{array}{c} P_{1}=P_{2}+\frac{1}{2}\rho \left(v_{2}^{2}-v_{1}^{2}\right) \end{array}$$

In Figure 6, the *P*_1_, pressure was measured at the pressure transducer; *P*_2_, pressure at the valve; ρ, density of the fluid; *v*_1_, fluid velocity at the tube input; and *v*_2_, fluid velocity coming out of the valve. From Equation 13, the value of *P*_1_ represented the value of the constant pressure measured after the repriming pressure. Given that the trapped air was exhausted at this stage, the constant pressure after repriming should be equal between the air (+) and air (−) conditions, and, in fact, our results agreed with the theory.

**Figure 7.**
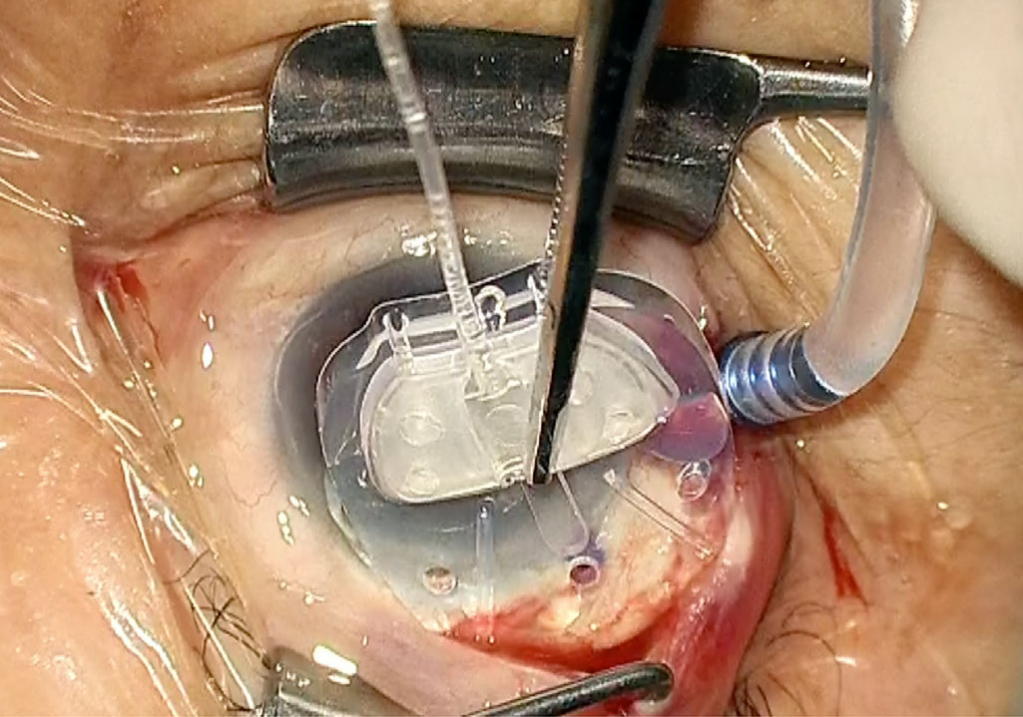
The surgical findings of the AGV implantation. After the initial priming, entrapped air bubbles are seen in the device tube intra-operatively.

Intra-operatively, after the initial priming, air can be trapped in the AGV tube when the needle is pulled out of the tube (Fig. 7). To avoid the unintended postoperative pressure rise (although the rise may be transient), the authors recommend that surgeons avoid leaving the air in the tube intra-operatively. Another translational relationship of our observation is that using the pars plana tube insertion combined with gas tamponade surgery, such as when treating neovascular glaucoma,^17^^,^^18^ may result in higher IOPs than expected during the early postoperative days. Conversely, use of air/gas can be a good way to avoid postoperative hypotony when the AGV is implanted in eyes with a high risk of hypotony, such as in the presence of high myopia, aphakia, uveitis, and in elderly patients.^18^^,^^19^

In addition to the results of this study at constant pressure after repriming air (-), we also compared the mean values of the new and old AGVs, which have almost the same mean pressure values. To avoid early postoperative hypotony, the manufacturer changed the inspection standards of the valve closing pressure in 2020. A concern may be that this change increases the final IOP level with new lots compared with old lots. However, because the constant pressures after repriming were equivalent between the old and new lots, we can expect that the final pressures postoperatively do not differ greatly between the current and older devices.

Considering that the current results were all derived from experimental settings, the clinical relevance remains to be proven. Therefore, translational use of the evidence requires particular attention for patients’ safety.

In conclusion, based on precise measurement of the flow characteristics, the presence of trapped air in the AGV tube increases the repriming pressure, whereas the air does not affect the constant pressure afterward.
